# ^R^NeuMark: A Riemannian EEG Analysis Framework for Neuromarketing

**DOI:** 10.1186/s40708-022-00171-7

**Published:** 2022-09-16

**Authors:** Kostas Georgiadis, Fotis P. Kalaganis, Vangelis P. Oikonomou, Spiros Nikolopoulos, Nikos A. Laskaris, Ioannis Kompatsiaris

**Affiliations:** 1 Centre for Research & Technology Hellas, Information Technologies Institute (ITI), Thermi-Thessaloniki, Greece; 2 AIIA-Lab, Informatics Dept, AUTH, NeuroInformatics.Group, Thessaloniki, Greece

**Keywords:** Neuromarketing, Riemannian Geometry, Covariance Matrices, Electroencephalography, BCIs

## Abstract

Neuromarketing exploits neuroimaging techniques so as to reinforce the predictive power of conventional marketing tools, like questionnaires and focus groups. Electroencephalography (EEG) is the most commonly encountered neuroimaging technique due to its non-invasiveness, low-cost, and its very recent embedding in wearable devices. The transcription of brainwave patterns to consumer attitude is supported by various signal descriptors, while the quest for profitable novel ways is still an open research question. Here, we suggest the use of sample covariance matrices as alternative descriptors, that encapsulate the coordinated neural activity from distinct brain areas, and the adoption of Riemannian geometry for their handling. We first establish the suitability of Riemannian approach for neuromarketing-related problems and then suggest a relevant decoding scheme for predicting consumers’ choices (e.g., willing to buy or not a specific product). Since the decision-making process involves the concurrent interaction of various cognitive processes and consequently of distinct brain rhythms, the proposed decoder takes the form of an ensemble classifier that builds upon a multi-view perspective, with each view dedicated to a specific frequency band. Adopting a standard machine learning procedure, and using a set of trials (training data) in conjunction with the associated behavior labels (“buy”/ “not buy”), we train a battery of classifiers accordingly. Each classifier is designed to operate in the space recovered from the inter-trial distances of SCMs and to cast a rhythm-depended decision that is eventually combined with the predictions of the rest ones. The demonstration and evaluation of the proposed approach are performed in 2 neuromarketing-related datasets of different nature. The first is employed to showcase the potential of the suggested descriptor, while the second to showcase the decoder’s superiority against popular alternatives in the field.

## Introduction

Neuromarketing is an emerging field that interconnects neuroscience and consumer behavior studies with economics [1, 2]. As a concept, it is originated by the need of researchers and practitioners in the field to gain a more objective overview about consumers’ decisions and preferences and the belief that there are fragments of information that are unobtainable by traditional marketing practices, like focus groups, questionnaires, interviews, and behavioral metrics [3]. These practices that are in principle behavioral and subjective have been successfully embodied in the field of marketing research since they are characterized by low-cost, scalability, and easy/swift interpretations. Nevertheless, the main identified drawback is the lack of generalizability that in turn results in questionable reliability in terms of predictive power [3]. Additionally, there are several occasions that the participants’ responses have been identified as inaccurate, unreliable, biased, and in the case of focus groups even affected by other participants’ opinions [4].

The transition from conventional marketing to neuromarketing is achieved via the incorporation of neuroimaging techniques, which are employed to examine the brain’s physiological responses to advertisement-related stimuli. Out of the various available neuroimaging methods, electroencephalography (EEG) is the one encountered in the majority of neuromarketing-based studies, as it is non-invasive, portable, can be obtained at a relatively low cost, and provides measurements of high temporal resolution. The lower spatial resolution anticipated in EEG when compared to other neuroimaging technologies is “compensated” by EEG’s previously described characteristics. In essence, neuromarketing is a typical example of a passive Brain–Computer Interface (BCI) [5], as the gathered neurophysiological responses are used to monitor the user’s cognitive states (e.g., attention, mental workload, memorization) and not as an alternative communication or control pathway, which is the case for active BCIs [6].

EEG-based neuromarketing studies identify and exploit different cognitive processes depending on the study’s question(s) and objectives. Approach–withdrawal is probably the dimension of cognitive processes analyzed in the majority of them, as it indicates whether the participant is attracted (approach) or not (withdrawal) to a particular stimuli (e.g., commercial advertisement, product) [7–9]. In essence approach–withdrawal is an index, usually referred as AW, that quantifies the hemispheric asymmetry in the prefrontal cortex, i.e., it estimates the difference in terms of brain activity between the left and right prefrontal brain area filtered in alpha frequency band (α; 8–13 Hz). A relatively higher left frontal activation usually translates to a positive AW and indicates the approach phenomenon, whereas an increase in the right frontal activity usually reflects a negative AW and is indicative of the withdrawal phenomenon [10]. Similarly to the AW, there are some studies that formulate the choice index by examining the frontal asymmetric beta (β; 13-30 Hz) and gamma (γ; 30–45 Hz) oscillations [11, 12]. Another cognitive process that affects the decision-making process and as a result is encountered in a plethora of neuromarketing studies is mental workload [13-15]. Mental workload can be interpreted as the effort invested by consumers while making decisions (e.g., purchase or not a product), with the cognitive process being characterized by increased theta activation (θ; 4–8 Hz) in the prefrontal and frontal areas. Additionally, there are some studies that describe mental workload as a synchronization/desynchronization phenomenon [16], with the former referring to the process previously described and the latter to a decrease in alpha activity in the parietal lobe. Attention index [17, 18] is another cognitive index that is studied with respect to the decision-making process, since focusing to something implies that a selection/prioritization mechanism has been activated. Alternatively, consumers’ attention and engagement are evaluated at a population level, using inter-subject correlation [19, 20]. Within the same context, the memorization process [21], highly affects the consumers’ purchase habits since it is more likely to select a familiar product rather than a relatively unknown one. Additionally, consumers’ decisions are highly influenced by emotions; therefore, the cognitive task of emotional processing is considered interconnected with the decision-making process [22]. This resulted in a series of studies dealing with the task of emotion recognition, as a means to unravel consumers’ emotional state [23, 24]. Finally, there are several studies that jointly examine the aforementioned indices and indicators, aiming to craft models of higher predictive power (e.g., [16, 19, 25, 26]).

The aim of this study was to exploit Riemannian geometry concepts [27, 28] so as to introduce a novel EEG-based decoder for detecting the consumers’ preferences. Riemannian approaches are built upon the fundamental concepts of Riemannian geometry that adheres to the notions of differential geometry. EEG signals are represented as sample covariance matrices (SCMs) that are measured entities scattered over a particular Riemannian manifold, this of symmetric positive definite (SPD) matrices [28]. The initial motivation of this work stems from the following facts: (i) Riemannian approaches alleviate a series of challenges encountered in the typical EEG analytic pipelines, like the ones previously described (e.g., AW, mental workload, etc.) that mainly arise from the inherent signal properties (e.g., non-stationarity, artifact contamination, and subject/session variability) [27], (ii) A series of Riemannian geometry concepts have been successfully incorporated in various BCI applications and in several cases have led to more effective brain decoding compared to traditional EEG signal analytic pipelines [29–32], (iii) Riemannian geometry concepts have been successfully employed to describe the coordination of different brain areas [28], since as previously described the decision-making process requires the synergy of different brain areas and brain states that when combined resulted in superior decoding schemes (e.g., [16, 19]), and (iv) Despite the rapid growth of the field, to the best of our knowledge, Riemannian approaches have not yet been examined within the context of neuromarketing.

The main hypotheses interwoven with this study were: (i) The suitability of Riemannian Geometry concept for decoding the consumers’ intentions in neuromarketing-related scenarios and (ii) The necessity to exploit more than one frequency bands in the sample covariance estimation for “reading” the consumers’ choices/decisions. Consequently, the contribution of this work is twofold. We first show the suitability of the sample covariance descriptor within the neuromarketing context. Then, we exploit Riemannian geometry so as to introduce a novel brain decoding scheme for detecting the consumers’ preferences. To this end, each EEG trial is represented as a spatial covariance matrix, properly re-aligned within the SPD manifold, that in essence encompasses the functional covariation between signals recorded at distinct (recording) sites. Multidimensional Scaling (MDS) acts on the inter-covariance distances among all available covariance pairs giving rise to feature vectors that are then fed to a support vector machine (SVM) that casts a prediction. The process is repeated, independently, for multiple EEG frequency bands (i.e., delta (δ) – gamma (γ)) so as to incorporate all possible brain rhythms associated with the cognitive processes that have been identified as significant to the decision-making process [33]. This results in the realization of an SVM ensemble, that accomplishes the final recognition of the consumers’ preferences, with the final ensemble’s response resulting from the decision-making rule of majority voting.

The proposed approach is demonstrated and validated based on two different EEG datasets that correspond to distinct neuromarketing scenarios. The first includes data from our own experiments, where participants evaluate advertisements of static content (i.e., image) and is used to showcase the expressiveness of covariance patterns when handled within a Riemannian geometry framework. The second dataset is a publicly available one [19], where participants rank commercials of dynamic nature (i.e., videos) and is employed to verify the decoder’s superiority against popular alternatives.

The remainder of this paper is organized as follows: Sect. 2 describes the methodology for formulating our ^R^NeuMark decoder, Sect. 3 presents the employed datasets and the preprocessing steps followed, Sect. 4 is dedicated to the obtained results, and Sect. 5 discusses the added value and limitations of the study and the future perspectives of this work.

## Methodology

### Riemannian geometry preliminaries

Given a single trial ${X}_{i} \in {\mathbb{R}}^{ S \times T}, i=1, 2, \dots {N}_{trials}$ with $S$ and $T$ denoting the number of sensors and temporal samples, respectively, that is characterized by the corresponding class label ${y}_{i}\in \{0, 1\}$, the SCM can be estimated as ${C}_{i}= {X}_{i}{X}_{i}^{T}/(T-1)$, leading to an $S\times S$ representation for each trial. The derived covariance-based representations are by definition SPD matrices, given that the recorded brain activity (i.e., the temporal samples; Τ) is sufficiently large to ensure the full rank property of the covariance matrix. SPDs reside on a Riemannian manifold denoted by ${\varvec{Sym}_{\varvec{S}}^{\varvec{+}}}$, which can be visualized as a hypercone in the $S(S+1)/2$ dimensional Euclidean space, that encompasses symmetric matrices associated only with positive eigenvalues. The Riemannian manifold can be described as smooth and real manifold that is associated with a Euclidean tangent space at every point ${\varvec{P\in Sy{m}_{S}^{+}}}$. Typically, in EEG-related studies, the aforementioned Riemannian manifold is endowed with the Affine Invariant Riemannian Metric (AIRM). Then, the inter-covariance distance between a pair $({C}_{i},{C}_{j})$ of SCMs on the Riemannian manifold can be calculated using the AIRM-induced geodesic distance [34] which is formulated as

1 $$\delta \left({C}_{i},{C}_{j}\right)={\Vert logm({C}_{i}^{-1/2}{C}_{j}{C}_{i}^{-1/2}\Vert }_{F}$$

with $logm(.)$ being the log-matrix operator and ${\Vert .\Vert }_{F}$ the Frobenius norm of the matrix [17].

The Riemannian distance (see Eq. (1)) can be employed to determine the center of mass (or geometric mean) for a given set of covariance matrices using the Karcher/Fréchet means [35]. The process boils down to the identification of a unique point in the Riemannian manifold that satisfies the minimization of the sum of squared AIRM distances for a set of SCMs:

2 $$\overline{B } = {argmin}_{P\in {\varvec{S}}{\varvec{y}}{{\varvec{m}}}_{{\varvec{S}}}^{+} } \sum_{i=1}^{{N}_{trials}}{\delta }^{2}({C}_{i},P)$$

with ${N}_{trials}$ denoting the number of SCMs and δ(^.^,^.^) referring to the Riemannian distance defined in Eq. (1), while the computation of $\overline{B }$ is based on the iterative process proposed by Bini and Iannazzo [36].

### Riemannian alignment

The previously described SCM representations may significantly vary among subjects or recording sessions in terms of relative placement over the Riemannian manifold. More specifically, the SCMs of each subject/recording often follow a similar distribution (with the rest ones), but are centered at a different location over the same manifold. This is actually a covariate shift phenomenon that may significantly deteriorate the performance of any machine learning algorithm. In this direction, Zanini et al. [37] proposed an alignment process operating in the Riemannian framework with the scope of eliminating the phenomenon of “mis-placed” SCMs that will in turn allow the development of competent classifiers. The proposed data transformation is considered pivotal as it re-aligns all data points (i.e., SCMs) around the same reference point, which in our case will be the identity matrix. Mathematically the alignment for each SCM is formed as

3 $${C}_{i}^{A} = {\overline{B} }^{-1/2}{C}_{i}{\overline{B} }^{-1/2}$$

with $\overline{B }$ being the center of mass for a set of SCMs identified by Eq. (2).

### A Riemannian-based decoder for neuromarketing in EEG signals

**Fig. 1:**
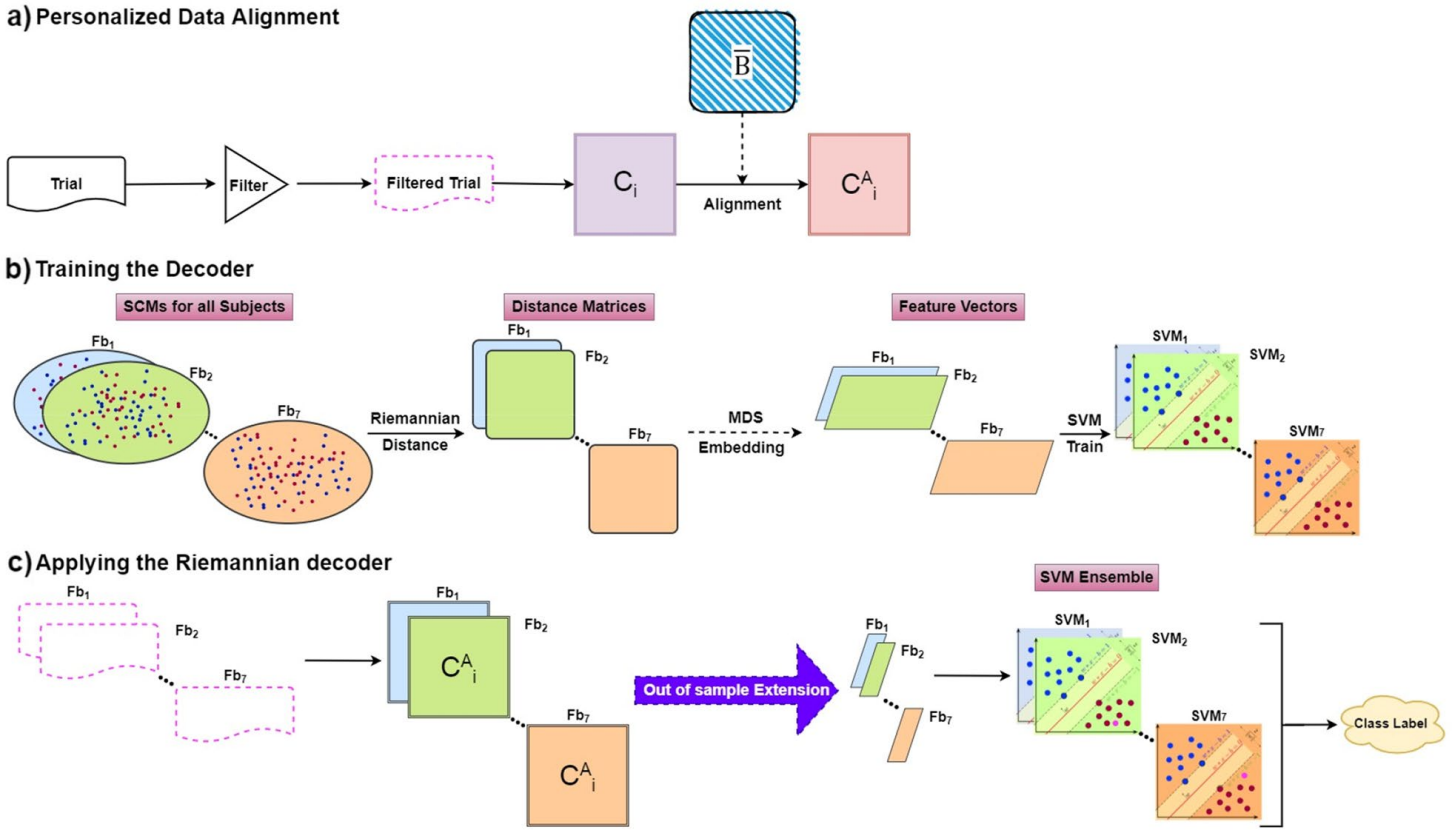
Flowchart of the ^R^NeuMark methodology

The proposed approach, denoted hereby as ^R^NeuMark, builds upon the previously introduced Riemannian geometry concepts with the scope of creating a robust pipeline for decoding the brain activity and consequently distinguishing the consumers’ preferred choices for various neuromarketing scenarios. The proposed pipeline is graphically illustrated in Fig. 1, with the first two panels (i.e., (a) and (b)) depicting the decoder’s designing process and the last (i.e., (c)) its application to unseen data. Here we assume that a train/test split of the trials is available, and both the following descriptions and illustrations of Fig. 1 refer to an instantiation of this split.

The initial point of our pipeline requires the band pass filtering of all EEG trials within a frequency band of interest. Then, the SCM for each trial is formulated as described in Sect. 2.1. The SCM derivation is followed by the SCM alignment (refer to Eq. (3)) that is performed in a personalized fashion (i.e., the estimation of the center of mass delivered by Eq. (2) is performed separately for each subject). Once the alignment process is completed the feature vector of each trial is constructed using MDS [38], a distance preserving dimensionality reduction technique, that acts on the Riemannian distances (see Eq. (1)) between all the available pairs of the re-aligned SCMs.

The decoder’s next step includes the incorporation of a classification scheme. Since SCMs are embedded, as vectors in a common Euclidean space (that approximates the corresponding SPD manifold), SVMs, that are known to provide efficient solutions for a wide range of brain activity-related problems [39], can be employed to discriminate among the consumers’ choices based on the re-aligned covariance representations. In the case of a binary classification task, SVMs’ training algorithm is formed to determine the hyperplane that can be characterized as optimal, i.e., the one that can both not only separate the two classes but also cope well with unseen data. The class association of an unseen trial (or covariance pattern) is dictated by the distance between the hyperplane and the trial. Here, the linear hyperplane is opted not only due to its low cost in terms of computational efficiency but also its established ability to provide efficient solutions.

The final step of the decoding scheme consists of the extension of the previously described computations to multiple frequency bands. This decision is dictated by the fact that the decision-making process encompasses various distinct brain states (e.g., approach/withdrawal and memorization) that are associated with different frequency bands [33]. For this study, seven commonly used EEG frequency bands (i.e.,${Fb}_{1}, {Fb}_{2}, ..., {Fb}_{7}$) were examined [40]: δ (1–4) Hz; θ (4–8) Hz; α1 (8–10) Hz; α2 (10–13) Hz; β1 (13–20) Hz; β2 (20–30) Hz; γ (30–45) Hz. In essence, the filtering step varies depending on the band limits, while the remainder of the computations are unaltered for each frequency band. In practice this leads to the formulation of an SVM ensemble consisting of seven distinct SVM models. The combination of the models’ predictions is based on the majority voting rule and the final decision regarding the label of any input trial is determined as the one encountered in at least four (out of seven) individual classifiers.

Finally, the application of the ^R^NeuMark decoder to an unseen trial requires the derivation of 7 re-aligned SCM representations and their placement in the corresponding band-specific learned embeddings prior to the activation of the SVM ensemble (refer to Fig. 1c). Τhe steps for deriving the aligned SCMs are in accordance with the ones presented in Fig. 1a. The process of incorporating a previously unseen SCM within a pre-learned embedding corresponds to an “*out of sample extension”* algorithm [38, 41, 42] and it is critical for the application of the trained model(s) to trials that have not been used in the initial training. This way, each unseen SCM residing in the SPD manifold [43] can now be efficiently formulated as a feature vector embedded in the identified by the training process low dimensionality setting [44] and can now be provided to each SVM in the ensemble.

## Experimental data and preprocessing

The efficiency and efficacy of the proposed approach are demonstrated experimentally, based on two EEG datasets captured under two distinct neuromarketing scenarios. The first dataset concerns the evaluation of advertisements of static content that was part of a preliminary study conducted in our laboratories and is employed as a means to validate the efficacy of the proposed descriptor. The second dataset is a publicly available one that was recently released by the authors of a neuromarketing-related study [19], concerns the ranking process of illustrations of dynamic content, and is used to establish the efficacy of the ^R^NeuMark decoder.

### Static content advertisements

**Fig. 2:**
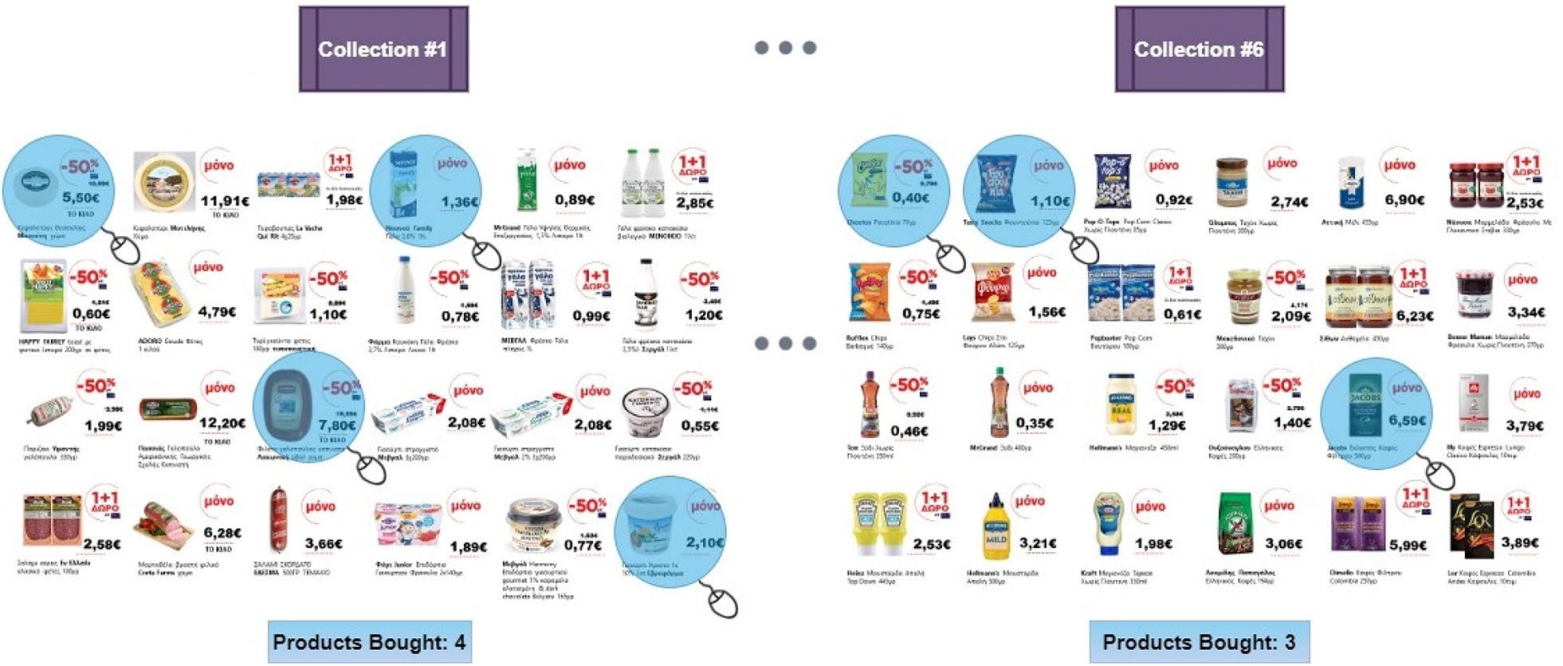
Experimental protocol for the static dataset. Six different image collections were delivered to the participant, who was allowed to select products from each collection without any restriction

Five individuals (3 males and 2 females, aged 34.83 ± 7.88), denoted as S1, S2, …, S5, participated in this study. Prior to the recording, subjects were thoroughly informed about the experimental procedure and gave written informed consent that was approved by the Ethical Committee of the Centre for Research & Technology Hellas (CERTH), with Ref. No. ETH.COM-68. Subjects were seated in a comfortable armchair placed 50 cm away from a 29-inch monitor and observed a series of image collections advertising supermarket products.^1^ In total 6 image collections, consisting of 24 different products each, were provided to the participants that could freely browse within them (by using the left and right arrow, respectively). Each image collection included products that belonged to the same product category (e.g., dairy, frozen, snacks, etc.). The task for the participants was to select (by left-clicking on) the products they intended to buy, without having any restrictions regarding either the time of observation for each collection or the total number of products being bought. The only “constraint” had been the instruction to perform these selections in accordance to their regular buying habits. This resulted in an uneven distribution among the trials of the two recording conditions, labeled as “buy” and “no-buy,” respectively. Figure 2 illustrates two such image collections, with the highlighted products indicating an exemplar case of selected products for each collection, while information regarding the total number of products bought in each collection is provided in the lower part of the figure.

The brain activity was recorded, with a sampling frequency of 500 Hz, via Neuroelectrics’ Enobio headset using an eight-sensor configuration. The selected sensors, namely, Fp1, Fp2, F3, F4, CP5, CP6, O1, and O2, were arranged according to the 10–10 International System, while prior to the experimental procedure impedance for all electrodes was set bellow 10KΩ. Finally, eye movements were captured via Tobii Pro Fusion eye tracker, with a sampling frequency of 600 Hz. They were used to define trials regarding the observation of the individual products, as intervals of stable eye fixations (refer to Sect. 3.3).

### Dynamic content advertisements

As a means of further validation, we utilized an additional and publicly available dataset. A total of thirty-one healthy individuals (13 males, aged 19–41) participated in this study. Three presentations of the same video commercial (i.e., dynamic content) for each of the six selected food products were delivered to the participants in randomized order. The length of each video commercial was between 25 and 46 s. Once the video presentation was completed, a product ranking was derived, using binary choice trials. Here, the classification task boils down to the discrimination of the participants’ first and last choices in terms of ranking that can be easily associated with the decision-making process and consequently the intention to buy (or not) a product. Finally, the encephalographic activity was registered, with a sampling frequency of 500 Hz, using Neuroelectrics’ StartStim 8, with the eight sensors, namely, F7, Fp1, Fpz, Fp2, F8, Fz, Cz, and Pz, being placed mainly in the prefrontal/frontal brain areas. The interested reader is referred to the publication that accompanies the dataset for a more detailed description of the experimental process [19].

### Preprocessing

Depending on the dataset, the definition of a single trial is different. For the static dataset a single trial is defined based on the time interval spent by the participant on each product image. The time spent can be easily deduced by the eye tracking metrics and is equivalent to the time the participant’s gaze was located within the boundaries of each product image. In the case of the dynamic dataset each trial consists of the samples in time that the participant was watching a specific video commercial registered by the corresponding number of sensors.

The offline preprocessing consisted of two stages. The first concerned the application of a wide band filter, where EEG signals were filtered within [0.5–45] Hz via a 3rd-order Butterworth filter (applied in zero-phase filtering mode). The second stage removed artifacts (usually arising from eyes, muscles or cardiac pulse), using a semi-supervised procedure based on independent component analysis (ICA) and adaptive filtering. More specifically, we took advantage of the wavelet-ICA denoising method [45] and followed a series of steps in order to suppress the artifacts in the recorded EEG signals: (i) Split each continuous multichannel signal into non-overlapping segments (10 s long), (ii) Apply ICA on each segment separately, (iii) Identify the artifact-related ICs (as in FORCe [46]), based on their statistical characterization according to kurtosis and skewness and the visual inspection of their spectra, (iv) Correct those ICs that had been identified as containing artefactual activity, using wavelet decomposition based on biorthogonal wavelets and wavelet shrinkage with a hard threshold based on false discovery rate [47], and (v) Reconstruct the multichannel signal from the denoised ICs (including the non-artifactual ones) and use the reconstructed signals for the proposed framework. All the reconstructed signals were further visually inspected ensuring the validity of this approach.

In the static dataset both steps were performed in the continuous EEG traces prior to trial segmentation (i.e., in the whole recording) aiming to avoid edge effects. In the dynamic dataset they are performed on a single-trial level since the dataset is provided with a given segmentation.

## Results

### Sample covariance descriptor in static advertisements

First, we demonstrate the validity of the sample covariance descriptor using the static dataset. Working for each subject independently, and after removing all trials shorter than 1 s (which did not convey sufficient information regarding delta band activity [48]), we derived all single-trial covariance patterns and compared against each other based on the Riemannian distance.

**Fig. 3:**
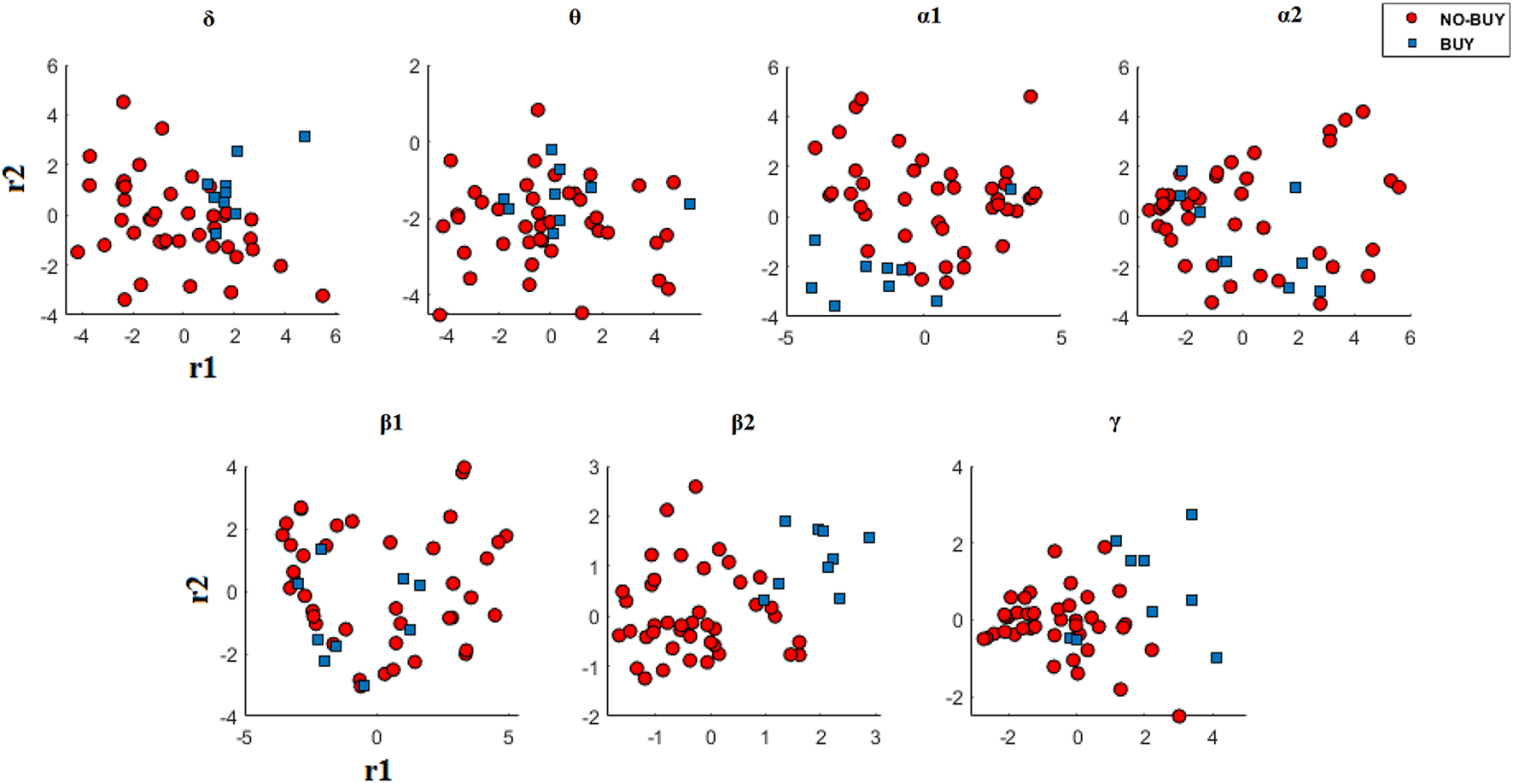
Brain rhythm-dependent semantic geodesic maps [38] of the single-trial covariance patterns relating to static advertisements in case of subject S5

Figure 3 graphically illustrates the MDS-based representations of these patterns as 2D points, for an indicative example of this dataset (i.e., subject S5). It can be seen that the derived representations for this subject are characterized by discriminability in more than one frequency bands (with β2 being the most prominent example, followed by γ, α2, and δ), revealing the necessity to examine multiple frequency bands that incorporate various cognitive states (e.g., mental workload is anticipated in θ band while approach withdrawal in α).

**Fig. 4:**
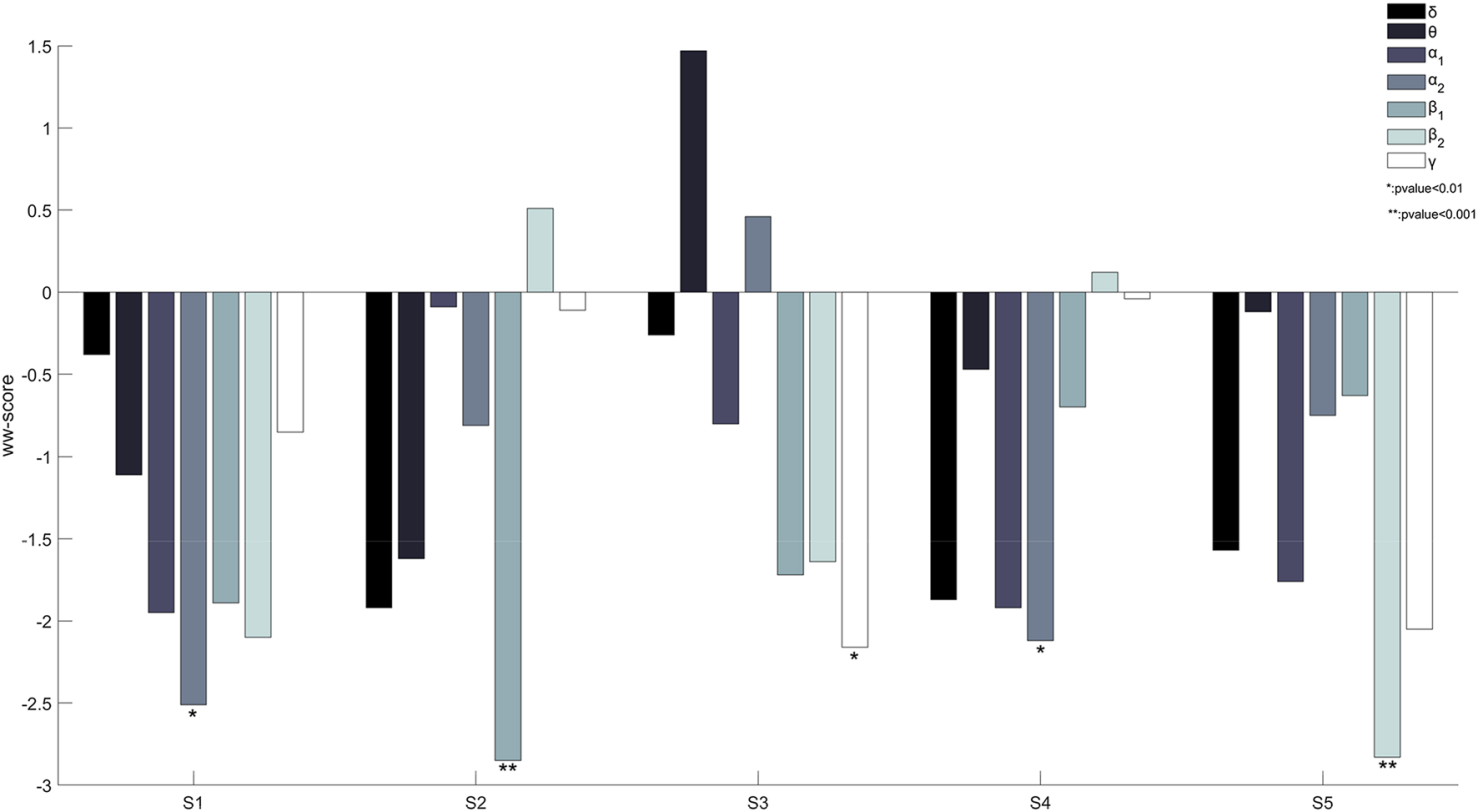
The obtained ww-scores for the static dataset. Low ww-score levels indicate high separability between “buy” and “no-buy” brain state

The trends observed in Fig. 3 align well with the scores obtained using an extension of the Wald-Wolfowitz test (WW-test), namely the multivariate WW test [49]. The multivariate WW-test was selected as a means to statistically compare the two recording conditions (i.e., “buy” and “no-buy”) based on the reduced MDS-representation of the associated covariance patterns, due to its intrinsic characteristics (i.e., nonparametric, generalizability) that align well with the unbalanced nature of the dataset. Returning to the specifics of WW-test, the lower the obtained score, called hereafter ww-score, the highest the separability among the two classes regarding a specific representation. For example, by visually inspecting the MDS embedding for subject S5 in β2 band (illustrated in Fig. 3), an almost clear separation among the data points of the two classes is observed with the ww-score being − 2.83 (the trend is statistically significant at a *P*-value of 0.001). On the contrary, in θ band (where the corresponding data points are entangled) the ww-score is − 0.12. Similarly, for the patterns of the other frequency bands previously characterized as discriminative (i.e., γ, α2, and δ) the obtained ww-score ranges between − 1.57 and − 2.05. Figure 4 includes the obtained scores for every subject and across frequency bands. It is evident that the lowest ww-score varies among subjects in terms of frequency bands and strength, and that there are cases in which more than one frequency band are associated with scores of high separability.

### Decoding dynamic advertisements

Next, we validate the sample covariance representation in the form of a fully developed decoder in the dynamic scenario, where it was utilized to discriminate between the participants’ highest and lowest ranked products. The validation protocol followed here is the one proposed by the dataset’s authors [19] so as to produce results that would be directly comparable. More specifically, a train/test split of 85%–15% was performed with the prerequisite that all views from a selected product are either included in train or test set. The reported results were obtained via repeating the train/test split process 10,000 times and estimating the averaged (across splits) classification performance along with the corresponding standard deviation.

**Fig. 5:**
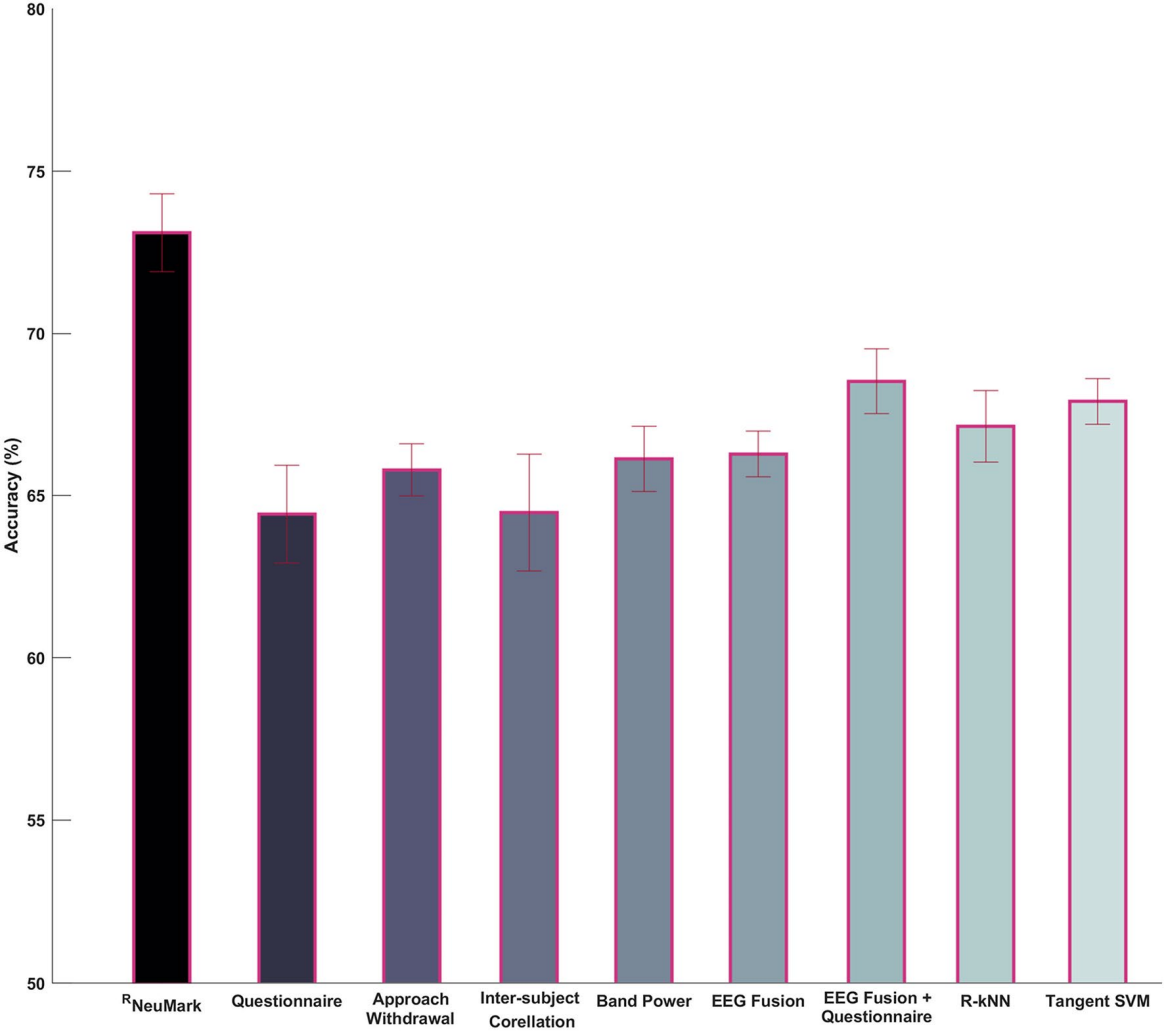
Classification performance for the decoders of users’ preference in the case of dynamic advertisements

Figure 5 illustrates the classification accuracy for the binary task of discriminating the highest/lowest-ranking product when the proposed decoder is employed. Additionally, it provides a direct comparison with popular neuromarketing metrics, namely, a conventional marketing approach (i.e., questionnaire), alternative neuromarketing EEG indices (i.e., approach withdrawal, inter-subject correlation, band-power, and their fusion) and the combination of the fused EEG indices with the questionnaire responses. It is important to mention here that three of the previously described metrics (i.e., questionnaire, EEG fusion and the EEG fusion + questionnaire) were also examined by the authors of the dataset [19], with the approach of *EEG fusion* + *questionnaire* being identified as the better performing one. Moreover, two popular classification procedures operating within the Riemannian framework were also examined [28, 50], namely, the R-kNN (Riemannian k-nearest neighbor) and the Tangent Space SVM, with the former examining the geodesic distances between covariance matrices under the scope of the classical kNN classifier, and the latter performing the classification task of SCMs in the (Euclidean) tangent space delineated by the barycenter of all the SCMs. Both approaches were tailored to the specifics of the ^R^NeuMark decoder (refer to Sect. 2.3) aiming to fair comparisons.

By visually inspecting Fig. 5, it is evident that the proposed decoding scheme yields a significantly improved performance (73.11%) compared to both the questionnaires (64.42%) and the various neuromarketing indices (63.22%—66.27%), that in some cases reaches a 10% improvement, with the differences being statistically significant at a P-value of 0.001. Additionally, it is worth noticing that ^R^NeuMark also outperforms the combined version of EEG and questionnaire features (68.51%), that is in line with the expected added value of neuro-indicators in the field of marketing. This trend is statistically significant at a P-value of 0.01 when using the *t*-test, with an effect size of 1.23, being estimated using Cohen’s d formula [51]. Finally, the ^R^NeuMark decoder outperformed also both the R-kNN (67.12%, *P*-value < 0.01) and the Tangent Space SVM (67.89%, *P*-value < 0.01), a trend that showcases the benefit of the introduced MDS embedding.

## Discussion

Riemannian geometry concepts have been widely explored by the neuroscientific community, with the information rich SCM representations providing valuable insights regarding brain functionality. The continuously increasing attention in Riemannian geometry is directly connected to the fact that it addresses the majority of the problems (e.g., non-stationarity, artifact contamination, and subject/session variability) encountered in classical signal processing algorithms resulting in more reliable decoding pipelines. The incorporation of such feature representations to the domain of EEG signal processing has led to robust classification schemes characterized by high predictive power concerning various classification problems. Nevertheless, despite their efficiency and popularity Riemannian approaches have not yet been exploited in neuromarketing-related problems.

In this work we examine the efficacy of the sample covariance representation and we present a novel decoding scheme for the appraisal of consumers’ choices based on the Riemannian geometry. Considering that there is not a conclusive answer regarding the cognitive states involved in the decision-making process and that these can differ among subjects, we decided to explore the SCM representations built upon EEG traces filtered in a set of frequency bands. Preliminary results regarding the static dataset (see Fig. 3) confirmed our original hypothesis, indicating the need to seek brain patterns (i.e., activations) in several frequency bands and not only within a single band. Based on the above, the training process of the ^R^NeuMark decoder is realized separately for each frequency band resulting in seven independent SVMs acting upon the MDS representations derived by the inter-covariance distances. Finally, the application of the decoder to previously unseen data includes, besides the multiple SCM formulation (i.e., one SCM per frequency band), the embedding of the unseen data points of high dimensionality in the data setting of low dimensionality formulated in the training process via the technique of out-of-sample extension. The expressiveness of the sample covariance descriptor was first demonstrated in the static dataset, where a clear separation between the two classes is observed in different frequency bands among subjects. Additionally, the static dataset also acts as a means of validation regarding one of our original hypotheses, i.e., that the decision-making process encompasses various distinct brain states interconnected with different brain rhythms, with Fig. 3 and Fig. 4 clearly indicating the variety of the optimal frequency bands among subjects. The descriptor’s efficacy when incorporated in a Riemannian geometry-aware decoder was validated in the dynamic dataset, with the proposed decoder offering improved classification accuracies compared to questionnaires, popular neuromarketing alternatives and classification schemes operating within the Riemannian framework (refer to Sect. 4). Here, probably the most noticeable observation is decoder’s superior performance compared to the combination of EEG and questionnaire features that can be characterized as statistically significant (*t*-test, *p*-value < 0.01).

One aspect of the present work that was left untreated and should be considered as a possible future extension is the transition to an online setting where advertisers could be informed about the effectiveness of their creations (e.g., product, packaging, commercial) in real time. This transition, that in essence completes a BCI system, requires a series of modifications since the current implementation regarding the decoding of unseen data comes at a complexity cost of $\mathcal{O}({S}^{3}N)$, with $\mathcal{O}({S}^{3})$ and $\mathcal{O}(N)$ being imposed by the AIRM [34] and out-of-sample extension [41] calculations, respectively. While linear complexity is acceptable for online BCI implementations, a cubic complexity could in some cases significantly hinder the online decoding process. More specifically, as $\mathcal{O}({S}^{3})$ is directly affected by the number of recording sensors (i.e., S), in recordings with a sparse sensor representation, which is the case for the validation datasets selected for this study, the computational cost can be characterized as affordable. On the contrary, in more dense sensor array configurations the execution time significantly increases. The most straightforward approach to resolve this issue is to decrease, in an efficient way, the number of recording sensors, and consequently the size of each SCM. One such approach could be the use of spatial filters [52], resulting in the selection of a predefined number of sensors. Alternatively, unsupervised approaches (e.g., [53, 54]) can be employed, aiming to identify the most informative sub-group of sensors that will be used in the formulation of each SCM.

Another potential extension of this study could be the conjunction of the introduced Riemannian aspects with the general theory of deep neural networks [55, 56] aiming to create a decoding scheme that yields even higher classification scores. Additionally, the noted benefits of the Individual Alpha Frequency (IAF) [51] to define the frequency ranges of the employed brain rhythms could be exploited toward identifying frequency ranges more fruitful for covariance pattern estimation than the standards ones. Within the same context, IAF approach is expected to be more useful in the case of personalized (or subject-specific) decoding schemes for neuromarketing. Moreover, alternative schemes could be examined in order to identify the most discriminant bands and employ only them in the subsequent classification task (e.g., [57]). Additionally, alternative automatic artifact removal/suppression techniques, like Artifact Subspace Reconstruction (ASR) [58] and FORCe [46], can be explored aiming in the removal of artifacts in real time. Finally, a particularly intriguing extension of the present study would be the exploration of the consumers’ incentive(s) behind the decision to purchase or not a product, given that appropriate information is collected via questionnaires. This would result in a multiclass classification problem (e.g., the decision was influenced by price, brand, discount etc.). While ^R^NeuMark decoder was introduced in a binary classification setting, the modification steps required to incorporate several classes seem feasible since SCMs will be encompassed in a common Riemannian manifold, while the remainder of the steps up to the SVM training will be unaltered.

## Data Availability

The dynamic dataset analyzed during the current study is available in the Dropbox, https://www.dropbox.com/sh/8kd1o2302d3i8gb/AAB2PRM6vJk_wtjstK8QU_I7a?dl=0. The static dataset analyzed during the current study is available from the corresponding author on reasonable request.

## Abbreviations

AW: Approach withdrawal
AIRM: Affine invariant Riemannian metric
BCI: Brain–computer interface
EEG: Electroencephalography
IAF: Individual alpha frequency
ICA: Independent component analysis
MDS: Multidimensional scaling
SCM: Sample covariance matrix
SPD: Symmetric positive definite
SVM: Support vector machine
WW-test: Wald-Wolfowitz test

## Footnotes

1 Images were provided by a local supermarket chain and the motivation was to replicate the layout of a standard advertisement leaflet.
